# Multicentric prospective study on computed tomography-guided periradicular infiltration and facet joint infiltration

**DOI:** 10.1007/s00234-025-03741-8

**Published:** 2025-08-16

**Authors:** Daniel Cantré, Lars Gerigk, Simon David Sprengel, Christian Plathow, Iris Burkholder, Marc-André Weber, Anna Schedler, Christoph Rehnitz

**Affiliations:** 1 Institute of Diagnostic and Interventional Radiology, Pediatric Radiology and Neuroradiology, University Medical Center Rostock, Rostock, Germany; 2 Department of Diagnostic and Interventional Radiology, Hufeland Clinics Bad Langensalza, Bad Langensalza, Germany; 3 Department of Oncology and Radiooncology, University Hospital Mannheim, Mannheim, Germany; 4 Radiologie Baden-Baden, Baden-Baden, Germany; 5 Department of Health Sciences, Fulda University of Applied Sciences, Fulda, Germany; 6 Department of Diagnostic an Interventional Radiology, University Hospital, Heidelberg, Germany

**Keywords:** Periradicular infiltration, Facet joint infiltration, CT-guided interventions, Back pain, Treatment effects

## Abstract

**Purpose:**

Evaluation of the success of periradicular infiltration and facet joint infiltration in a multicenter and prospective approach.

**Materials and methods:**

114 patients undergoing therapeutic nerve root or facet joint infiltration for radicular and/or facet joint symptoms between the first lumbar and the first sacral segments were prospectively and consecutively enrolled across nine participating study centers in Germany. These centers provide CT-guided pain procedures continuously over a period of 96 months. Assessment was carried out by means of a systematic patient survey including pain questionnaires. The severity of the pain and the impairment caused by pain were assessed using 11-point Numerical Rating Scales.

**Results:**

CT-guided periradicular infiltration and facet joint infiltration significantly reduced pain levels and substantially reduced pain frequency at all time points and up to three months after the last intervention. All pain related characteristics (i.e. actual pain, average pain level, maximum pain level) were significantly (95% confidence intervals (CI) ranging between − 1.0 and − 5.0) reduced at all time points when compared to the baseline. Besides the pure pain levels, the presented data also provide detailed insights into the complex associated issues the patient collective is facing including impairment in everyday life, fitness for work and leisure time activities. These parameters were also substantially improved (95% CI between − 0.0 and − 5.0) at all time points indicating a comprehensive patient benefit. Self-reported patient satisfaction, recommendation of the procedure and personal success were rated high (62–100%). The rate of patients on sick leave dropped from 23.7 to 1.9%. Accordingly, patients reported a high rate of satisfaction with the procedures. There was a high technical and clinical success rate with no major complications.

**Conclusion:**

CT-guided pain therapy on the lumbar spine is effective and safe with a comprehensive benefit for patients, including not only pain levels in the narrow meaning but also regarding everyday life, fitness for work and leisure time activities at all investigated time points including 3 months follow-up.

## Introduction

Back pain, i.e. lumbago and radiculopathy are common causes of pain-related limitations in the ability to work and quality of life and cause high societal costs through loss of ability to work and utilization of the health care system [1, 2]. Mechanical irritation of the nerve roots by degenerated intervertebral discs, vertebral bodies, vertebral joints, or ligaments is primarily responsible for the radicular pain symptoms, while isolated back pain is often caused by osteoarthritis of facet joints [3]. In periradicular infiltration (PRI) and facet joint infiltration (FJI) therapy, local anesthetics are injected around the affected nerve roots, and/or facet joints, respectively, in order to achieve acute pain relief and thereby enable conservative therapy of the underlying causes, e.g. by means of physiotherapy. In addition, corticosteroids are administered locally as part of PRI and FJI, which are intended to reduce swelling and inhibit local inflammatory reaction, thereby reducing mechanical and biochemical nerve root irritation, or arthritis, respectively. PRI is a widely used approach for managing sciatic pain, as it often leads to a subjective improvement in symptoms. Similarly, FJI is a common treatment for localized back pain, known for its relatively low rate of complications [4]. Although both methods are recommended under specific circumstances in orthopedic guidelines [5], aided by growing evidence through randomized controlled studies [6–12], there is a lack of prospective data on their efficacy in real-world medical care in Germany.

The aims of this prospective multicenter study are to further improve the scientific basis for the widespread use of PRI for sciatic pain and FJI for local back pain symptoms, and to assess their efficacy in daily practice within the German healthcare system. Notably, the study did not intend to address a comparison between different imaging-guiding modalities and intervention techniques but evaluate outcomes and safety using the most widely used method in the participating radiology centers, which was CT-guided intervention. For this purpose, both the immediate effect of the therapy and the further course of the pain symptoms up to three months after the end of the infiltration therapy were monitored. Using questionnaires, the quality and intensity of pain, as well as patient satisfaction following treatment, were systematically assessed at four time points: immediately prior to treatment initiation, shortly after treatment commencement, immediately before treatment completion, and three months post-completion of PRI or FJI therapy.

## Materials and methods

The study was performed according to the Declaration of Helsinki in its present form. Ethical approval was given by the Internal Review Board (Ethical Approval Code S-578/2013). Written and oral informed consent was obtained from all participants prior to inclusion into this prospective study.

Inclusion criteria were approved indication for the referred intervention according to applicable guidelines and good clinical practice, and age over 18 years.

Exclusion criteria were lack of informed consent, signs of septic arthritis and age under 18 years.

120 patients referred to the 9 participating study centers for CT-guided therapeutic nerve root or facet joint infiltration for radicular and/or facet joint symptoms between the first lumbar and the first sacral segments were prospectively and consecutively included continuously over a period of 96 months.

Assessment was carried out by means of a systematic patient survey including pain questionnaires, before the start, after the first therapeutic infiltration, before the end and three months after the end of therapy, focusing on the pain quality and quantity, functional and activity limitations pre- and post-intervention, and patient satisfaction after the treatment. The questionnaires included open questions, multiple choice items, as well as pain scales at multiple time points [13, 14]. Information on gender, age, height and weight was collected at baseline, and the body mass index (BMI) was calculated. Information on previous treatment and pain history was collected from all patients included in the analysis population at baseline. It was outside of the scope of this study to verify the specifics of the previous treatments as well as possible comorbidity, e.g. through investigation of medical records. The current pain intensity, the average recent pain intensity and the maximum pain intensity during the last 4 weeks were evaluated using a Numerical Rating Scale (NRS), i.e. a 11-point scale where zero represents no pain, 1 represents the lowest, and a value of 10 represents the worst pain imaginable [15]. Whenever multiple response options were selected on the scale, the higher value, representing the worst-case scenario, was recorded. Whenever the current pain intensity exceeded the maximum pain intensity, or the average pain intensity exceeded the maximum, the maximum pain intensity was adjusted to reflect the corresponding value. An identical NRS was used to assess pain-related impairment (0 = no impairment; 10 = total impairment). The effect of PRI and/or FJI was recorded at survey time point “2 – after the first therapy”. In the form of questionnaires, questions were asked about the improvement in pain, the current intensity of the pain, the side effects and satisfaction with the therapy. At the survey time points “2 – after the first therapy”, “3 – before the last therapy” and “4–3 months after the end of the therapy” the side effects, the recommendation of the therapy, the success of the therapy and the start of physiotherapy were recorded.

### Pretreatment

Possible adverse effects were evaluated in the form of open questions, and the narrative answers were categorized in accordance to an adverse event classification by the Society of Interventional Radiology [16].

**Fig. 1:**
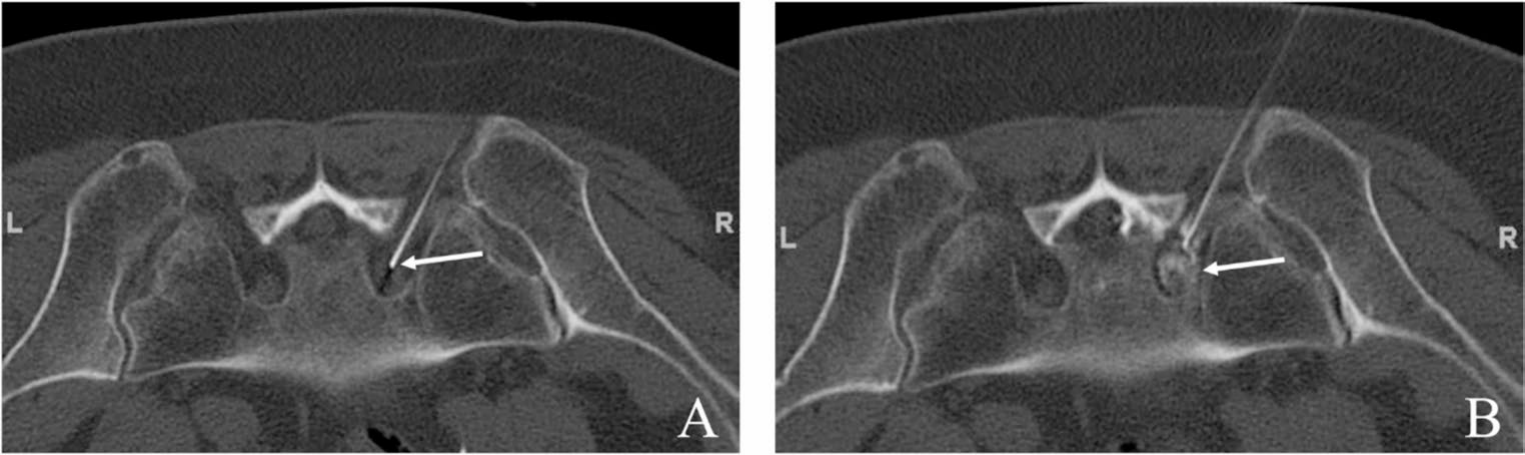
Typical example of CT-guided periradicular infiltration of the S1 nerve root on the right hand side of a 74-year-old female patient, performed in prone position. (**A**) final needle position before drug administration, focusing on the slice where the needle tip (white arrow) is positioned directly adjacent to the S1 spinal nerve in the neuroforamen. (**B**) final CT-documentation to prove proper periradicular distribution of the drug cocktail (local anesthetics and cortical steroids mixed with iodinated contrast medium, white arrow)

**Fig. 2:**
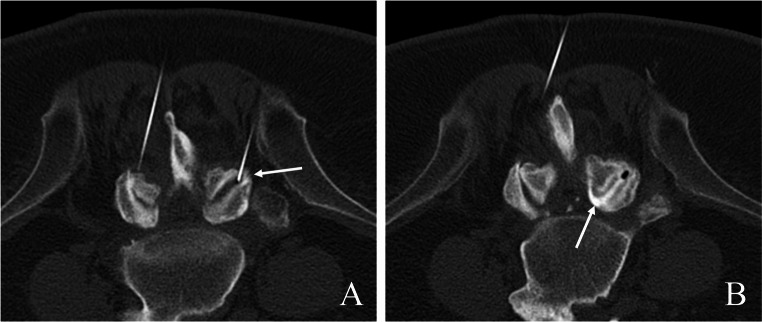
Typical example of simultaneous bilateral facet joint infiltration L4/L5 in a 56-year-old male patient, performed in prone position. (**A**) documentation of final needle position intraarticularly within the right facet joint L4/L5 (white arrow). (**B**) final CT-documentation for the right facet joint, proving proper intraarticular distribution of the drug cocktail (local anesthetics and cortical steroids mixed with iodinated contrast medium, white arrow)

To guarantee an adequate level of competence, all procedures were performed by board certified radiologists with a minimum of six years of experience in the method applied. Procedures were carried out according to national and international guidelines [17–20], with CT-documentation of the final needle positioning before the drug administration as well as drug distribution post infiltration, see Figs. 1 and 2.

In the case of PRI, a small amount of diluted iodinated contrast medium was injected before drug administration, to rule out intravasal or intradural injection and to ensure epidural periradicular spreading of the drugs. The typically applied drug cocktail contains 3 ml bupivacaine hydrochloride 0,5% (Carbostesin) and 1 ml triamcinolone acetonide (Volon A 40 mg/ml). The CT protocol using different multidetector scanners was adjusted on the individual patient anatomy and focused on desired treatment level or segment. A typical CT protocol on a Siemens SOMATOM Emotion 16 scanner using 130 kilovoltage (KV) and 90 milli Ampere seconds (mAs), included a focused topogram of the region of interest (for instance lower lumbar spine, 0.6 mm slice thickness (ST), dose-lengths-product (DLP) 16 mGy*cm) and focused spiral scans of the treatment segment (one planning, 1 needle positioning, 1 control after drug administration) with 3 mm ST and DLP of about 42 mGy*cm per scan). Typical calculated median radiation doses were below 3 mSv [21], which lies within the typical range of CT-guided therapies [19].

#### Statistics

Statistical analysis was carried out using the SAS 9.4 software (SAS Institute Inc., North Carolina, USA). For continuous parameters, the arithmetic mean and the standard deviation, as well as the median, minimum and maximum values were reported, while for categorical variables, absolute and relative frequencies were presented.

The severity of the pain and the impairment caused by pain were assessed using 11-point NRS. These were evaluated descriptively with statistical parameters for each time point assessed. The deviations from the baseline values were calculated, with negative deviations indicating an improvement and positive deviations indicating a decline compared to the baseline. These deviations were also analyzed descriptively. Furthermore, empirical distribution-free 95% CI were determined for the median differences from baseline values.

Additionally, for explorative post-hoc comparison of FJI versus PRI, two groups were built with patients receiving one of these treatment options exclusively. Patients receiving a combination of both treatments and individuals with equivocal information on treatment targets were excluded from the post-hoc analysis. Comparative statistical analysis was carried out using nonparametric statistical tests, where applicable. P-values of < 0.05 were regarded as statistically significant.

## Results

Of the 120 eligible individuals screened, six had to be excluded because of missing informed consent (*n* = 4) or missing or faulty information in the case report form (*n* = 2). 114 patients (pts) (55 male (48.2%), 54 female (47.4%), undisclosed gender information in 5 individuals (4.4%) with a median age of 59 years (22 to 81 years of age), a mean height of 172.7 ± 9.81 cm, mean body weight of 81.9 ± 15.90 kg with a resulting BMI of 27.4 ± 4.80 kg/m^2^, could finally be included in the study. Of 114 individuals included, 37 (32.5%) received FJI and 41 (36%) received PRI exclusively, 16 patients (14.0%) received a combination of FJI and PRI, and in 20 individuals (17.5%) the treatment target was not clearly specified, although within inclusion criteria. Participation in the surveys declined over the course of the study. From 100.0% (114 pts) before therapy, participation was 99.2% (113 pts) after the first treatment, 61.4% (70 pts) before the last treatment and 46.5% (53 pts) three months after therapy. Data on the number of necessary treatments are available from 82 patients. These patients received a median of three interventions (minimum 1 and maximum 8). Demographic data and patient characteristics are summarized in Table 1.

**Table 1:** Demographics and patient characteristics

		total *n* = 114
**sex**	not provided	5 (4.4%)
	male	55 (48.2%)
	female	54 (47.4%)
**age (years)**	n	108
	mean	58.5
	SD	12.64
	median	59.0
	min	22.0
	max	81.0
**height (cm)**	n	113
	mean	172.7
	SD	9.81
	median	173.0
	min	150.0
	max	196.0
**weight (kg)**	n	114
	mean	81.9
	SD	15.90
	median	82.5
	min	45.0
	max	134.0
**BMI (kg/m²)**	n	113
	mean	27.4
	SD	4.80
	median	26.8
	min	18.9
	max	44.6

Note – n: number, SD: standard deviation, min: minimum, max: maximum

### Types of pain pretreatments

Only 10 patients (8.8%) reported that they had not received any prior treatment from healthcare professionals. The patients received a median of 2 pretreatments from different medical professions (minimum 1 and maximum 9). Of the 104 patients with previous treatment, 71.2% (74 pts) consulted an orthopedist, 57.7% (60 pts) a general practitioner and 26.9% (28 pts) a radiologist. The heterogeneous group of professions providing previous treatment is shown in Table 2.

**Table 2:** Distribution of professions providing previous treatment

	total *n* = 104
**Psychiatrist**	2 (1.9%)
**Surgeon**	4 (3.8%)
**Traditional healer**	6 (5.8%)
**Internal specialist**	8 (7.7%)
**Neurosurgeon**	10 (9.6%)
**Other**	13 (12.5%)
**Pain therapist**	15 (14.4%)
**Psychotherapist**	17 (16.3%)
**Neurologist**	18 (17.3%)
**Radiologist**	28 (26.9%)
**General practitioner**	60 (57.7%)
**Orthopedist**	74 (71.2%)

Note – n: number

### Lumbar spine surgery

A total of 9 patients (7.9%) stated that they had not received any prior pain treatment. Four patients (3.5%) did not answer this question. The patients received a median of 2 different prior treatments (minimum 1 and maximum 10). Of the 101 patients with previous pain treatment, 83.2% (84 pts) received pain therapy by means of per oral analgesics, 52.5% (53 pts) physiotherapy, and 50.5% (51 pts) injections/nerve blocks. Patients did neither disclose frequency nor the exact date of the last pain pretreatment before study inclusion.The distribution of all types of previous pain treatments is shown in Table 3.

**Table 3:** Distribution of pain treatment modalities

	total *n* = 101
**multimodal pain therapy**	1 (1.0%)
**relaxation techniques**,** hypnosis**,** biofeedback**	3 (3.0%)
**others**	6 (5.9%)
**physical rehabilitation**	7 (6.9%)
**infusions**	9 (8.9%)
**chiropractic**	11 (10.9%)
**acupuncture**	13 (12.9%)
**psychotherapy**	14 (13.9%)
**massages**	22 (21.8%)
**injections**,** nerve blocks**	51 (50.5%)
**physiotherapy**	53 (52.5%)
**analgesic drugs**	84 (83.2%)

Note – n: number

82.9% of patients (87 pts) stated that they had not yet had spinal surgery, while 12.4% (13 pts) had already had spinal surgery. This question was not answered by 4.8% of participants (5 pts). Of the 13 patients operated on, 10 specified the number of previous operations, the median number is 2 (minimum 1 and maximum 5). The specific localization of the operation was reported by 12 patients. Two patients stated that they had undergone surgery on the cervical and thoracic spine. One patient underwent surgery exclusively on the cervical spine, while nine patients had procedures solely on the lumbar spine.

### Past pain history

The patients were also queried regarding the duration of their pain and whether a specific event could be identified as its trigger. Among the respondents, 21.2% (24 pts) reported a pain duration of 1 month to 6 months. Pain persisting for 6 months to 1 year was noted by 18.4% of patients (21 pts), while 17.5% (20 pts) indicated that the onset of their pain occurred 2 to 5 years prior.

Data regarding the exact onset date of pain was available for 25.4% of patients (29 pts), while 19.3% (22 pts) reported the occurrence of a specific triggering event associated with the onset of pain. A pension application was submitted by 7.9% (9 pts). The median pain duration over the past 4 weeks was 28 days, with a range from 1 to 30 days. When asked for a desirable treatment goal, patients regarded a pain level of 2 (0 to 9) on the 11-point NRS as tolerable. The main results are summarized in Table 4.

**Table 4:** Pretreatment pain characteristics

		total *n* = 114
**duration of pain**	< 1 month	16 (14.0%)
	1 to 6 months	24 (21.1%)
	6 to 12 months	21 (18.4%)
	1 to 2 years	16 (14.0%)
	2 to 5 years	20 (17.5%)
	> 5 years	17 (14.9%)
**exact onset of symptoms**	missing	3 (2.6%)
	yes	29 (25.4%)
	no	82 (71.9%)
**trigger existent**	missing	1 (0.9%)
	yes	22 (19.3%)
	no	91 (79.8%)
**pension application**	missing	9 (7.9%)
	yes	9 (7.9%)
	no	96 (84.2%)
**duration of pain within last 4 weeks**	n	94
	mean	25.1
	SD	7.21
	median	28.0
	min	1.0
	max	30.0
**tolerable pain level**	n	112
	mean	2.4
	SD	1.90
	median	2.0
	min	0.0
	max	9.0

Note – n: number, SD: standard deviation, min: minimum, max: maximum

### Pain surveys

All characteristics related to pain frequency and duration are presented separately for each survey time point in Table 5.

**Table 5:** Pain characteristics survey

		pre treatment *n* = 114	after 1 st treatment *n* = 98	before last treatment *n* = 70	3 months after treatment *n* = 53
**frequency of pain**	missing	2 (1.8%)		2 (2.9%)	3 (5.7%)
	multiple times daily	104 (91.2%)	51 (52.0%)	21 (30.0%)	25 (47.2%)
	once daily	1 (0.9%)	20 (20.4%)	11 (15.7%)	5 (9.4%)
	multiple times weekly	3 (2.6%)	9 (9.2%)	7 (10.0%)	5 (9.4%)
	once weekly	1 (0.9%)	5 (5.1%)	12 (17.1%)	2 (3.8%)
	multiple times monthly		1 (1.0%)		3 (5.7%)
	once monthly	1 (0.9%)			
	< once per month	2 (1.8%)	12 (12.2%)	17 (24.3%)	10 (18.9%)
**duration of pain**	missing	8 (7.0%)	9 (9.2%)	7 (10.0%)	7 (13.2%)
	seconds	5 (4.4%)	13 (13.3%)	18 (25.7%)	4 (7.5%)
	minutes	17 (14.9%)	46 (46.9%)	28 (40.0%)	16 (30.2%)
	hours up to 3 days	34 (29.8%)	19 (19.4%)	10 (14.3%)	16 (30.2%)
	> 3 days	50 (43.9%)	11 (11.2%)	7 (10.0%)	10 (18.9%)
**employment**	missing	2 (1.8%)	1 (1.0%)		2 (3.8%)
	yes	50 (43.9%)	47 (48.0%)	35 (50.0%)	29 (54.7%)
	no	62 (54.4%)	50 (51.0%)	35 (50.0%)	22 (41.5%)
**sick note**	missing	1 (0.9%)	2 (2.0%)		2 (3.8%)
	yes	27 (23.7%)	22 (22.4%)	13 (18.6%)	1 (1.9%)
	no	86 (75.4%)	74 (75.5%)	57 (81.4%)	50 (94.3%)

Note – n: number

Prior to treatment, the majority of patients experienced pain multiple times per day (104 pts, 91.2%), with pain episodes lasting for several hours to more than three days (84 pts, 73.7%). Following the initial treatment, the proportion of patients experiencing pain multiple times daily and for durations of several hours to more than three days decreased to 52.0% (51 out of 98 pts) and 30.6% (30 out of 98 pts), respectively. Prior to the final treatment, these proportions were further reduced to 30.0% (21 out of 70 pts) and 24.3% (17 out of 70 pts), respectively. Three months after the final treatment, 47.2% (25 out of 53 pts) of patients reported experiencing pain multiple times daily, and 50.1% (26 out of 53 pts) reported pain lasting several hours to more than three days. The percentage of patients with pain less than once per month increased ten-fold from 1.8% (2 out of 114 pts) to 18.9% (10 out of 53 pts). Prior to treatment, 43.9% (50 out of 114 pts) of patients reported being actively employed, while 23.7% (27 out of 114 pts) were on sick leave at the time of the survey. Three months after the last treatment, the rate of employment was 54.7% (29 out of 53 pts), with only 1.9% (1 out of 53 pts) on sick leave.

The severity of the pain was assessed at each time point of the survey. The evaluation of the individual parameters at the individual survey times is summarized in Table 6.

**Table 6:** Absolute pain intensities

		1 – pre treatment *n* = 114	2 - after 1 st treatment *n* = 98	3 – before last treatment *n* = 70	4 − 3 months after treatment *n* = 53
**present pain intensity**	n	113	97	70	49
	mean	6.6	4.6	3.4	3.7
	SD	2.57	2.30	2.43	2.85
	median	7.0	5.0	3.0	4.0
	min	0.0	0.0	0.0	0.0
	max	10.0	9.0	8.0	10.0
**recent pain intensity**	n	112	96	69	48
	mean	7.0	4.8	3.9	4.4
	SD	1.87	2.24	2.34	2.40
	median	7.0	5.0	3.0	5.0
	min	0.0	0.0	0.0	0.0
	max	10.0	9.0	8.0	10.0
**maximum pain intensity**	n	113	97	70	50
	mean	8.4	5.9	5.3	6.6
	SD	1.68	2.35	2.74	2.52
	median	9.0	6.0	4.5	8.0
	min	0.0	0.0	0.0	0.0
	max	10.0	10.0	10.0	10.0

Note – n: number, SD: standard deviation, min: minimum, max: maximum

**Fig. 3:**
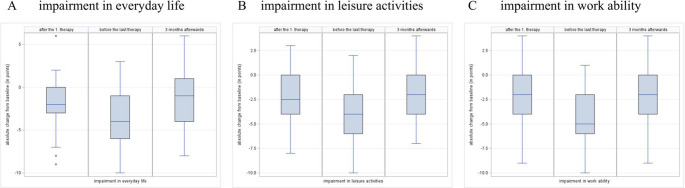
Change of pain levels reported by the patients using an 11-point numerical rating scale (0 = no pain, 1 = lowest and 10 = worst pain imaginable) after the first treatment, before the last treatment and 3 months afterwards, compared to baseline values before treatment. (**A**) change of pain levels at the respective survey time points. (**B**) change of mean pain levels over the last four weeks before the respective survey time points. (**C**) change of the maximum pain levels within the four weeks before the respective survey time points

In addition, the difference from the baseline value before the start of therapy was taken into account, as visualized in Fig. 3.

Before treatment, the median intensities reported for the pain at the present moment, as well as the average pain in the four weeks prior was 7.0/10. Both reduced to 5/10 after the first treatment and to 3/10 before the last treatment. Three months following the last treatment, the median present pain intensity was reported as 4.0/10, while the average recent pain intensity was 5.0/10. Maximum pain intensity was also positively affected, with a median of 9/10 before treatment, 4.5/10 before the last treatment, and 8.0/10 three months after therapy. When compared to baseline, maximum therapy effect was found at the survey time point before the last treatment, with a reduction of 4.0 points for present as well as average recent pain intensity (95% CI −5.0 to −2.0), and 3.0 points for maximum pain intensity (95% CI −5.0 to −2.0). Three months post-therapy, median pain intensities remained reduced by a mean of 2.0 points for both present and average recent pain (95% CI −4.0 to −1.0), and by 1.0 point for maximum pain intensity (95% CI −2.0 to −1.0) (Fig. 3). As zero is not included in the 95% confidence intervals at every time point and for all parameters, it can be concluded that there is a significant reduction in pain intensity in all parameters and at every time point compared to baseline.

### Pain-related impairment

The impairments in everyday life, leisure activities and the ability to work were considered. The statistical parameters are shown in Table 7.

**Table 7:** Pain related impairment

		pre treatment *n* = 114	after 1 st treatment *n* = 98	before last treatment *n* = 70	3 months after treatment *n* = 53
**everyday life**	n	112	95	65	49
	mean	5.9	4.2	3.2	3.9
	SD	2.80	2.29	2.34	2.69
	median	6.0	4.0	3.0	4.0
	min	0.0	0.0	0.0	0.0
	max	10.0	9.0	9.0	9.0
**leisure activities**	n	111	96	65	51
	mean	6.6	4.4	3.4	4.3
	SD	2.67	2.50	2.31	2.62
	median	7.0	4.5	3.0	5.0
	min	0.0	0.0	0.0	0.0
	max	10.0	10.0	9.0	9.0
**fitness for work**	n	112	96	63	51
	mean	6.5	4.5	3.4	4.3
	SD	2.96	2.55	2.50	2.56
	median	7.0	4.0	3.0	5.0
	min	0.0	0.0	0.0	0.0
	max	10.0	10.0	10.0	9.0

Note – n: number, SD: standard deviation, min: minimum, max: maximum

**Fig. 4:**
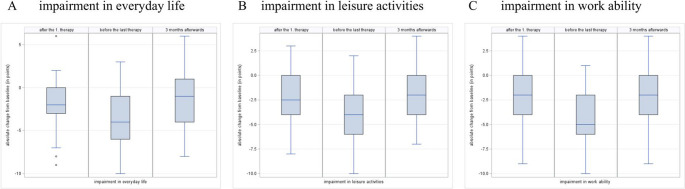
Change of impairment levels reported by the patients using an 11-point numerical rating scale (0 = no impairment, 1 = lowest and 10 = total impairment) after the first treatment, before the last treatment and 3 months afterwards, compared to baseline values before treatment. (**A**) change of impairment levels regarding everyday life activities at the respective survey time points. (**B**) change of impairment regarding leisure activities. (**C**) change of impairment regarding the ability to work

In addition, the difference from the baseline value before the start of therapy was considered as visualized in Fig. 4.

Throughout the course of therapy, pain-related impairment showed the greatest improvement at the time point preceding the final treatment, with a median reduction of 4.0 points in all three categories (95% CI −5.0 to −3.0). At three months after therapy, improvement persisted by 1.0 point for everyday life (95% CI −2.0 to 0.0), and 2.0 points for both leisure activities and fitness for work (95% CI −3.0 to −1.0). Except for the impairment in everyday life at the last observation time point 3 months after the end of therapy zero is not included in the 95% confidence interval at all other time points and for all other parameters. It can be concluded that there is a significant reduction in pain-related impairment for these parameters and the respective time points compared to baseline.

### Treatment efficacy

The evaluation of all questions about therapy are summarized in the following Table 8.

**Table 8:** Treatment effect

		after 1 st treatment *n* = 98	before last treatment *n* = 70	3 months after treatment *n* = 53
**side effects**	missing	2 (2.0%)	1 (1.4%)	2 (3.8%)
	yes	17 (17.3%)	12 (17.1%)	11 (20.8%)
	no	79 (80.6%)	57 (81.4%)	40 (75.5%)
**recommendation**	missing	5 (5.1%)	1 (1.4%)	2 (3.8%)
	yes	90 (91.8%)	65 (92.9%)	40 (75.5%)
	no	3 (3.1%)	4 (5.7%)	11 (20.8%)
**success**	missing	9 (9.2%)	3 (4.3%)	6 (11.3%)
	yes	80 (81.6%)	62 (88.6%)	33 (62.3%)
	no	9 (9.2%)	5 (7.1%)	14 (26.4%)
**physiotherapy started?**	missing	2 (2.0%)		2 (3.8%)
	yes	33 (33.7%)	36 (51.4%)	24 (45.3%)
	no	63 (64.3%)	34 (48.6%)	27 (50.9%)

Note – n: number

Throughout the course of therapy, about 17% of patients (17 out of 98 pts) subjectively reported side effects, this percentage seemingly increased to about 21% three months after therapy (11 out of 53 pts). It is important to note that, in the questionnaires, patients were instructed to indicate the presence of suspected side effects using checkboxes (yes or no) and to provide a narrative description of any adverse events in their own words. A total of 30 different patients reported side effects at different time points, not all of them consistently, leading to 45 different observations. Three patients answered the question only at time point 4, three months after end of therapy, and retrospectively added descriptions of events encountered at a previous survey time point. For further interpretation we first excluded observations that were obviously part of the intended treatment, i.e. anesthesia in the dermatome of the treated nerve root (n = 3). Most side effects reported were typical, expectable mild adverse events known to frequently occur with PRI and FJI, especially when corticoids are added, and patients had been informed about pre therapy, such as cortisone flush (reported 14 times) and short periods (< 24 h) of palsy corresponding to the nerve root treated. Interestingly, some patients reported side effects closely related to the therapeutic procedure exclusively in retrospect at the last survey 3 months after therapy, explaining an ostensible increase at the last time point. Two individuals consistently reported a side effect resembling a cortisone flush at each survey time point, including time point 4, where no treatment was administered. One of these individuals described a ‘reddening of the face’ as an allergic reaction to corticosteroids at time point 3, but reclassified it as a cortisone flush at time point 4. Additionally, one individual reported an ‘allergic reaction’ (burning sensation in face) at time point 2, but did not report any further adverse events in subsequent surveys. This is interpreted as a description of a typical cortisone flush. Two individuals reported worsening of pain. One of them additionally described heart burn, paresthesia of the left foot, and bilateral gluteal muscle ache, with worsening of the symptoms and additional “renal problems” at 3 months after therapy. The other one reported weak and trembling legs and described paresthesia in both legs and bilateral palsy for about 3 months, that returned to nearly normal at time point 4, three months after the last therapy. The remaining adverse events reported were rash at one arm (1), nose bleed (1), drowsiness/dizziness (3), nausea (4), fatigue and sweating (1), high blood pressure (1), weight gain before last therapy (2), weight loss after first therapy (1), headache (3), xerostomia (1), deterioration of oral microflora (1), and vasovagal reaction (2). Not all of these narratives can be attributed to the procedures themselves. Nevertheless, all these reported side effects were classified as mild adverse events, Class 1 according to the SIR AE severity score [16], and no additional treatment or unplanned hospital admissions were needed.

Prior to their final treatment, 88.6% (62 out of 70 pts) of patients considered the treatment successful, and 92.9% (65 out of 70 pts) indicated they would recommend the treatment to others. Three months post-treatment, 62.3% (33 out of 53 pts) of patients still considered the treatment successful, and 75.5% (40 out of 53 pts) would recommend the procedure. Following the first treatment, only one-third (33 out of 114 pts) of patients were able to initiate physiotherapy. This proportion increased to over 50% before the final therapy session and remained approximately 45% (24 out of 53 pts) three months after the final procedure.

### Accompanying pain medication

Data on concomitant pain medication were collected through open-ended questions in the questionnaires at each survey time point. Since this information was not coded, no statistical analyses can be carried out. There was inconsistency in the reporting of the specific drugs, often inadequate information on the dosage, and only a minority of patients gave information on pain medication at every time point. Consequently, we are limited to providing a narrative summary of certain observations. Of 74 patients that reported their medication before the first treatment, 64 were given NSAIDs, and 19 were on opioids. In 38 patients, data on pain medication from the pre-interventional visit and at least one visit after the first treatment was available. Among these, 19 patients experienced a reduction in the frequency and/or dosage of oral pain medication. Of the four who were using opioids, two were able to discontinue them entirely, while the remaining two managed to reduce their opioid doses. Seventeen patients reported no change in their pain medication, with four of them remaining on opioids. Two patients, who stated they were on opioids but not NSAIDs before interventional treatment, reported increased opioid doses. One only gave information at survey time point 2, and information on pain medication after the end of therapy is missing. The remaining patient reported initiating NSAIDs three months after the final intervention.

### Post-hoc group comparison

As participation in the study declined over time, the sizes of the two subgroups were relatively small, as presented in Table 9.

**Table 9:** Group sizes for post-hoc comparison of FJI versus PRI

	FJI	PRI
pre treatment	37 (100.0%)	41 (100.0%)
after first treatment	30 (81.1%)	34 (82.9%)
before last treatment	18 (48.6%)	26 (63.4%)
three months after treatment	22 (59.5%)	18 (43.9%)

Note: FJI = facet joint infiltration, PRI = periradicular infiltration. Number of patients per group and study time point. Percentages within groups relative to the pre treatment group size

For the vast majority of parameters compared, there were no substantial differences between the two groups for any parameter. The most important findings are summarized as follows. Median age was 60 years (39 to 80 years of age) in the FJI group and 57 years (22 to 81 years of age) in the PRI group (Mann Whitney U test: *p* = 0.34). Pain duration before study inclusion was reported as less than one month in 10.8% (4 pts) in the FJI group versus 22.0% (9 pts) in the PRI group, and as more than five years in 18.9% (7 pts) in the FJI group versus 4.9% (2 pts) in the PRI group (Chi square test *p* = 0.248). Only two parameters revealed statistically significant differences, however, only at one time-point in the treatment course each. Namely, maximum pain intensity after the first treatment was reported higher in patients receiving FJI with a median of 7 (minimum 2 and maximum 9) and a mean of 6.8 ± 1.99 compared to PRI with a median of 6 (minimum 1 and maximum 10; Mann-Whitney-U test, *p* < 0.05) and a mean of 5.7 ± 2.08. Three months after the last treatment, maximum pain intensities were reported as a median of 8.0 (minimum 1 and maximum 10) and a mean of 6.9 ± 2.11 in the FJI group versus a median of 6 (minimum 0 and maximum 10) and a mean of 6.6 ± 2.81 in the PRI group (*p* = 0.98). Patients receiving FJI reported significantly more side effects (5 pts, 27.8%) compared to FJI (1 pt, 3.8%) before the last treatment (Fisher’s exact test, *p* < 0.05). Three months later side effects were noted by 22.2% (4 pts) in the PRI group and 18.2% (4 pts) in the FJI group (*p* = 1.0).

## Discussion

This prospective multicenter study underlines the efficacy of CT guided PRI and JFT in patients with lumbago and lumboischialgia, through significant reduction of pain levels, substantial reduction of pain frequency, and improvement of pain-related impairment, with enduring positive effects up to three months after the last intervention.

Patients in the current cohort experiencing lower back pain were primarily middle-aged to older adults, with a predominance of individuals exhibiting overweight or class 1 obesity. While age is among the main unadjustable risk factors, obesity represents the main adjustable risk factor for lumbar degenerative disease [22] and may be adequately addressed through lifestyle changes and especially fought by physical activity [23]. However, when lumbar degenerative disease becomes symptomatic, resulting in pain-related functional impairment and the development of kinesiophobia, a vicious circle may ensue. In this context, achieving adequate pain relief becomes a crucial prerequisite for the successful implementation of long-term therapeutic strategies [24].

Most patients experienced chronic, frequent or persistent, and severe lower back and/or sciatic pain, with no identifiable trigger or clear onset date. Approximately half of the patients were actively employed, highlighting the significant impact of chronic lower back pain on workforce productivity, causing high societal costs, in addition to its adverse effects on daily life and leisure activities. This is underlined by nearly 24% of employed patients being on sick leave at the time of the pre-treatment visit. Conversely, less than 8% of patients were seeking pension benefits related to chronic pain, suggesting a strong preference to remain active within the workforce whenever feasible. PRI and FJI demonstrated significant efficacy in reducing the proportion of patients on sick leave, with a reduction from 24% prior to treatment to less than 2% at three months post-treatment.

Only a small proportion of patients had undergone prior high-cost surgical interventions on the lumbar spine. However, patients sought a variety of different treatment options from multiple different health-care providers, mainly orthopedics, general practitioners and radiologists, prescribing analgesic drugs in over 80%, but physiotherapy and PRI/FJI in only about 50%. Despite having received a median of two different prior treatments for their symptoms, over two-thirds of patients continued to experience chronic back pain lasting from more than six months to over five years at the time of the initial study visit. Therefore, the efficacy of those previous therapy regimes seems questionable, while generating high ongoing costs for the healthcare system, further adding to the societal costs.

Most patients in our study initially suffered from multiple daily pain attacks or pain attacks lasting hours to more than three days, with a median recent pain intensity of 7/10, and median maximum pain intensity of 9/10 on an 11-point NRS. Interestingly, when asked for acceptable treatment outcome, patients did not expect total pain relief but regarded a pain level of 2/10 tolerable.

Treatment with PRI and/or FJI resulted in a significant reduction in median present and average recent pain levels by 2.0 points following the initial treatment and by 4.0 points prior to the final intervention, while peak pain intensity decreased by 3.0 points prior to the final procedure. Three months after the last treatment there was still a reduction by 2.0 points of median present and average pain levels, and by 1.0 points of peak pain events. Pain-related impairment improved by up to 4.0 points over the course of therapy and remained improved by 2.0 points for leisure activities and work fitness at three months following the final treatment. The portion of patients with very high frequency (> 90% of pts) and long lasting (> 70% if pts) pain attacks dropped to approximately 50% at three months post-treatment. These results on the efficacy of both CT-guided pain therapy methods in our study is in line with the results of other groups [25].

Patients did not expect to be pain free after therapy but rather wished for a significant pain reduction. Although achieving the patients’ goal of reducing pain intensity to 2/10 was unattainable in many cases, PRI and/or FJI significantly reduced pain severity and improved pain-related impairment, with these effects sustained for up to three months following the final therapy session. Accordingly, PRI and FJI were very well perceived by the participants. After the first treatment, over 80% of patients felt the therapy was successful, and more than 90% of patients would have recommended the therapy to others. Three months after the last treatment 2/3 of patients still regarded therapy successful, and 3/4 would recommend it to others. Also, a subset of participants experienced a reduction in pain medication usage. Notably, the proportion of employed patients on sick leave decreased significantly three months post-therapy. During the treatment course, over 50% of patients commenced adjunctive physiotherapy, which declined to 45% at three months following the final intervention, possibly due to the expiration of physiotherapy prescriptions. Nonetheless, this remained above the baseline level of 33%. In addition to the reduction of impairment in daily, leisure, and work activities, the restoration of physical activity capacity to facilitate weight loss represents a crucial component in the management of chronic back pain.

No major complications occurred. The adverse effects reported by participants, as detailed in their own descriptions, were largely consistent with those anticipated for PRI and FJI interventions, including transient paresis and cortisol flush. Additionally, some participants misinterpreted treatment outcomes, such as experiencing anesthesia-like sensations. Other adverse effects reported included headaches, mild vasovagal responses, and additional corticosteroid-related effects. One report of nosebleeds three months after the last therapy may theoretically represent a symptom of elevated blood pressure from steroid-induced arterial hypertension. This individual received a total of six PRIs including steroids. As we do not have blood pressure values before or after treatment, we cannot completely rule out steroid induced hypertension in this case. However, systemic drug effects are generally rare with local steroid application [26] and the patient reported the use of Ibuprofen in the form of self-medication, but no antihypertensive medication, rendering symptomatic arterial hypertension less likely. Another report of weight loss immediately after the first therapy session is unlikely attributable to the intervention. There were no reports of severe adverse effects requiring immediate medical intervention or hospitalization.

Additional explorative post-hoc subgroup analysis revealed no substantial, statistically significant differences between the two treatment options. While the proportional distribution of FJT and PRI-cases, respectively, was in line with epidemiological data in the literature [27], the group sizes of patients receiving exclusively FJI or PRI in our study were relatively small at the beginning and further declined over the course of the study, making comparative statistical analysis a challenging task. Although not statistically significant, there were a few patients of younger age in the PRI group compared to the FJI group, which is also reflected in the literature, as radicular pain is more likely the cause of lower back pain in younger patients, and incidence of facet joint arthritis is higher in the elderly [27]. There was also a tendency for long-lasting lower back pain in patients receiving FJI and a shorter period of pain in patients receiving PRI before study inclusion. This seems plausible, because of different clinical courses of the typical underlying conditions, i.e. facet joint arthritis as a chronic degenerative disease, and radicular compression that may occur without advanced lumbar spine degeneration in the case of acute lumbar disc herniation. Insignificant group differences may be explained by the small subgroup sizes, as well as an overlap in the pathophysiology, e.g. nerve root compression through intervertebral foramen stenosis caused by facet hypertrophy. Unfortunately, as this study relies on patient surveys rather than clinical reports or pre-treatment imaging data, we do not have sufficient information on the underlying pathophysiological conditions on which the treatment indications were based. However, with the already small subgroup-sizes, the inclusion of more discriminators would have further diminished statistical power, and it seems questionable that differences of efficacy regarding different causes of nerve root compression, e.g. bony foraminal stenosis, disc herniation or lumbar spinal canal stenosis would have proven significant. Regarding pain intensity, the benefit of PRI seems higher compared to FJI only for the maximum pain intensity, and only after the first treatment. We think this again reflects the underlying pathology, as pain caused by nerve root compression may be adequately addressed by mono-segmental therapy, whereas patients with symptomatic facet joint arthritis are more likely to suffer from multi-segmental disease, that cannot be treated in a single session. In the further treatment course, and especially three months after the treatment, no significant differences were found between the two groups. Interestingly, patients receiving FJI reported significantly more side effects before the last treatment, but there was no difference three months later. A possible explanation is that in three patients with FJI, involuntary indirect nerve root infiltration [28] caused unexpected radicular anesthesia, that the patients no longer reported as side effect retrospectively three months later.

### Limitations

Our study is an observational study on patients undergoing PRI and FJI. We have to acknowledge that our study has no control group without therapy or with sham procedures. Data collection in the form of patient surveys included open questions, leading to sometimes unfavorable data heterogeneity in these items. Comparative analysis of the different treatment groups was done as a post-hoc analysis and suffered from small subgroup sizes. Participants were only followed-up for 3 months following the last CT-guided intervention. The contributions of the various study centers were heterogeneous, and comparisons of the imaging-guided intervention techniques used across centers were not feasible.

## Conclusion

In conclusion, the CT-guided pain therapy methods PRI and FJI on the lumbar spine have been shown to be both effective and safe. These approaches result in significant improvements in pain intensity, frequency, and pain-related disability. Furthermore, patients experience sustained benefits, including improved physical activity and increased participation in both work and recreational activities, with effects lasting up to 3 months after the final treatment.

## Data Availability

Data is provided within the manuscript.
